# [Retracted] Mammalian STE20‑like kinase 1 regulates pancreatic cancer cell survival and migration through Mfn2‑mediated mitophagy

**DOI:** 10.3892/mmr.2024.13294

**Published:** 2024-07-22

**Authors:** Yongli Hu, Bing Wang, Lie Wang, Zhenran Wang, Zhiyuan Jian, Lin Deng

Mol Med Rep 22: 398–404, 2020; DOI: 10.3892/mmr.2020.11098

Following the publication of this paper, it was drawn to the Editors’ attention by a concerned reader that certain of the JC-1 staining images in Fig. 2C were strikingly similar to data appearing in different form in other articles written by different authors at different research institutes that had either already been published elsewhere prior to the submission of this paper to *Molecular Medicine Reports*, or were under consideration for publication at around the same time (a small number of which have been retracted). In addition, the Snail western blot data in Fig. 3E bore a close similarity to certain of the Mfn2 data shown in Fig. 4A. In view of the fact that certain of the contentious data had already apparently been published previously, and owing to a lack of confidence in the presentation of certain of the data in this paper, the Editor of *Molecular Medicine Reports* has decided that this paper should be retracted from the Journal. The authors were asked for an explanation to account for these concerns, but the Editorial Office did not receive a reply. The Editor apologizes to the readership for any inconvenience caused.

